# Sanguinarine Enhances the Integrity of the Blood–Milk Barrier and Inhibits Oxidative Stress in Lipopolysaccharide-Stimulated Mastitis

**DOI:** 10.3390/cells11223658

**Published:** 2022-11-18

**Authors:** Zhijie Zheng, Yonghui Zheng, Xiaoben Liang, Guanhong Xue, Haichong Wu

**Affiliations:** 1 Department of Veterinary Medicine, College of Animal Sciences, Zhejiang University, Hangzhou 310058, China; 2 Key Laboratory of Fujian Universities Preventive Veterinary Medicine and Biotechnology, Longyan University, Longyan 364012, China

**Keywords:** sanguinarine, mastitis, blood–milk barrier, oxidative stress, Nrf2, Wnt/β-catenin

## Abstract

Mastitis is a common clinical disease which threatens the welfare and health of dairy cows and causes huge economic losses. Sanguinarine (SG) is a plant-derived alkaloid which has many biological functions, including antibacterial and antioxidant properties. The present study attempted to evaluate the effect of SG on lipopolysaccharide (LPS)-induced oxidative stress reactions and explore its potential mechanisms. The expression profile of SG was analyzed by network pharmacology, and it was found that differentially expressed genes were mainly involved in the Wnt signaling pathway and oxidative stress through GO and KEGG enrichment. In in vitro experiments, the dosage of SG was non-toxic to mouse mammary epithelial cells (mMECs) (*p* > 0.05). SG not only inhibited the increase in ROS induced by LPS, but also enhanced the activity of antioxidant enzymes (*p* < 0.05). Moreover, the results of the in vivo experiments showed that SG alleviated LPS-induced inflammatory damage of mouse mammary glands and enhanced the integrity of the blood–milk barrier (*p* < 0.05). Further studies suggested that SG promoted Nrf2 expression and suppressed the activation of the Wnt signaling pathway (*p* < 0.05). Conclusively, this study clarified the protective effect of SG on mastitis and provided evidence for new potential mechanisms. SG exerted its antioxidant function through activating Nrf2 and inhibiting the Wnt/β-catenin pathway, repairing the blood–milk barrier.

## 1. Introduction

Mastitis is an epidemic in the global dairy industry, mainly caused by pathogenic microorganism infection, which can lead to the decline of milk production and quality [1,2]. In addition, mastitis can also lead to prolonged estrus and even the death of cows postpartum, which seriously threatens the welfare and health of cows and causes huge economic losses to humans [3,4]. Mammals during the peripartum period, which lasts from 3 weeks before to 3 weeks after parturition, are physiologically unstable and susceptible to a number of metabolic diseases compromising productivity [5,6].

It has been proved that many microorganisms can cause cow mastitis, among which *Escherichia coli* is one of the significant pathogenic microorganisms causing clinical mastitis [7]. Lipopolysaccharides (LSPs) in the cell wall of *E. coli* can cause inflammation and trigger innate immune responses, leading to a series of inflammatory reactions [8]. More and more evidence has shown that the Wnt/β-catenin signaling pathway is related to LPS-induced diseases, which cause the upregulation of inflammatory factors and lead to breast injury [9,10]. In addition, some studies have shown that LPS can also increase the production of reactive oxygen species (ROS) and change mitochondrial membrane potentials [11]. The blood–milk barrier, composed of mammary epithelial cells, is the most important line of defense in the protection of mammary glands [12]. The main structure of the blood–milk barrier is the tight junction (TJ), which forms a tight barrier that only allows the passage of small molecules and prevents the penetration of adjacent cell membranes [13,14]. LPS can destroy tight junction proteins after causing mastitis, leading to the degradation of the barrier, invasion of harmful substances and microorganisms, and aggravation of oxidative stress [15]. Oxidative stress in early-lactation cows exerts an important role in dysfunctional inflammatory response [16]. Therefore, it is particularly important to protect the integrity of the blood–milk barrier and suppress the pathogenic bacteria leading to excessive inflammatory response.

For many years, most dairy farms have mainly used antibiotics to treat mastitis, but over time the pathogens have developed drug resistance, and antibiotic residues in dairy products have become more and more serious, endangering human health [17]. In addition, vaccines for the treatment of bovine mastitis have not produced good results [18]. Therefore, there is an urgent need to find and develop new therapies for bovine mastitis.

Sanguinarine (SG), a plant-derived alkaloid, has many pharmacological functions, such as anti-oxidation, anti-inflammatory, and anti-tumor properties [19,20]. The results of animal experiments have suggested that SG relieved the symptoms of Dextran Sulfate Sodium (DSS)-induced colitis in rats [21]. However, it is not clear whether SG has a protective effect on LPS-induced mastitis. Therefore, we explored the role of SG in an LPS-stimulated mouse mastitis model and explored the possible mechanisms.

## 2. Materials and Methods

### 2.1. Reagents

**Figure 1:**
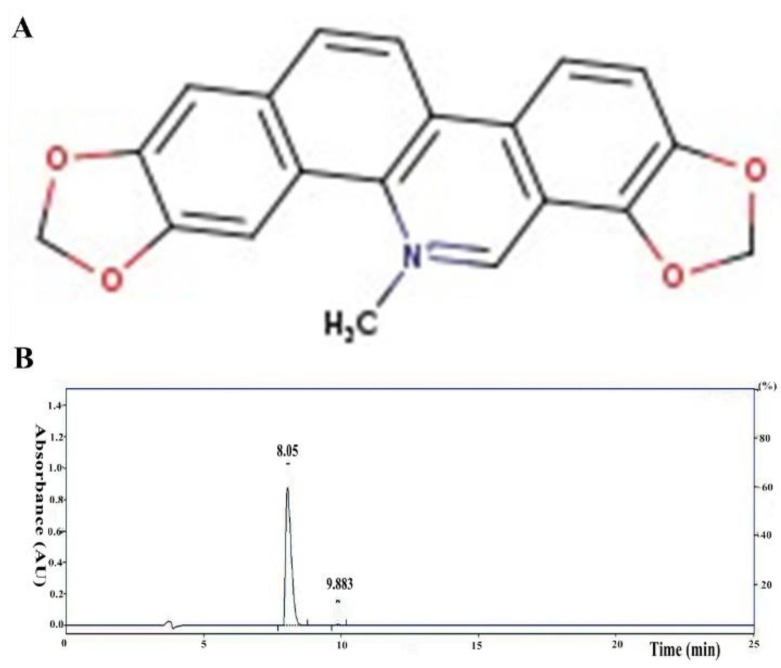
(**A**) The chemical structure of SG. (**B**) HPLC chromatogram of SG.

LPS was purchased from Sigma-Aldrich (055:B5, San Diego, CA, USA). The antibodies used in the experiments were purchased from Cell Signaling Technology (CST, Danvers, MA, USA). Sangui-narine (SG, purity ≥98%; Figure 1A) was obtained from Yuanye Biotech Co., Ltd. (Shanghai, China). The purity of the SG was detected by high-performance liquid chromatography (HPLC). The experiment was conducted on the EChrom2000 DAD Data System. Chromatography was performed with a SinoChrom 0DS-BP column (4.6 × 250 mm, 5 μm). An elute with 0.1% phosphoric acid water/acetonitrile at a flow rate of 1.0 mL/min was used, and detection with DAD at 325 nm was performed (Figure 1B). The ELISA kits used (for TNF-α and IL-1β) were purchased from Wuhan Boster Biological Technology, Ltd. (Wuhan, China).

### 2.2. Animal Treatment and Experimental Design

Sixty female BALB/c mice (8 weeks old, weighing 20–25 g) were purchased from the Animal Center of Zhejiang University. Before the experiment, all mice were given sufficient water and feed and stored in a 12/12 h dark/light-cycle environment. The whole feeding process was maintained at room temperature and 65% humidity. The animals were cared for humanely; all experiments involving the mice were conducted according to the Guide for the Care and Use of Laboratory Animals of the National Research Council, and all experimental protocols were followed by the Institutional Animal Care and Use Committee of Zhejiang University (approval number: GBT 35892-2018).

The mice were randomly divided into the following six groups: a control group, an LPS group, sanguinarine groups (SG groups: 5, 25, and 50 µM), and a dexamethasone group (5 mg/kg, DEX group). SG was dissolved and diluted in CMC Na (Sigma, San Diego, CA, USA) to give final concentrations of 5, 25, and 50 µM. The mouse mastitis model was prepared as described previously [22]. Briefly, one hour before the onset of LPS-induced mastitis, SG (5, 25, and 50 µM) or dexamethasone (5 mg/kg) was injected intraperitoneally twice every six hours. After pentobarbital anesthesia, LPS was injected into the two abdominal mammary glands for 24 h (the fourth pair of mammary glands, R4 and L4). Finally, the mice were sacrificed by CO_2_ inhalation, and the mammary tissues were collected for further study.

### 2.3. Histopathological Examination

The samples of mammary glands were fixed in 10% formalin. Paraffin sections were prepared by dehydration with graded alcohol. Next, the tissues were sectioned and stained with hematoxylin. Finally, the H&E-stained sections were observed under a light microscope, and images were collected to evaluate pathological changes.

### 2.4. Myeloperoxidase (MPO) Analysis

The mouse mammary gland tissue samples, weighing 100 mg, were ground in 2 mL PBS solution and centrifuged at 12,000 rpm for 15 min at 4 °C. Then, the supernatants were collected and analyzed using the MPO kit (Nanjing Jiancheng Biotechnology Co., Ltd., Nanjing, China). Finally, according to the calculation formula, the MPO enzyme activity of each sample was calculated.

### 2.5. Cell Culture and Treatment

As previously described, after collecting mammary gland tissues from the lactating mice, the digested tissues were suspended and passed through a cell filter to remove larger tissue debris. Epithelial cells were obtained by removing fibroblasts, endothelial cells, and other single cells. The isolated mMECs were cultured at 37 °C in a 5% CO_2_ humidified incubator containing 10% fetal bovine serum (FBS, Gibco, New York, NY, USA) supplemented with 100 U/mL penicillin and streptomycin and 10 μg/mL insulin. The mMECs were pretreated with different concentrations of SG (5, 25, and 50 µM) for 1 h and then stimulated with LPS (1 μg/mL) for 6 h.

### 2.6. Cell Biological Detection and Viability Assay

Cells were fixed with paraformaldehyde for 15 min at room temperature and washed three times with PBS. Cells were then blocked with 10% normal goat serum for half an hour at room temperature and incubated with primary antibody overnight at 4 °C. After the completion of primary antibody adsorption, the cells were incubated with fluorescent-labeled secondary antibodies (Bios, Beijing, China) for one hour at room temperature and washed three times in PBS. Nuclei were stained with Hoechst dye and then visualized with a laser scanning confocal microscope (Leica, Wetzlar, Germany).

Cell viability was determined using an MTT kit. Briefly, mMECs (1 × 10^5^ cells/mL) were passed in 96-well plates for 6 h and then treated with different concentrations of SG for 24 h. Finally, MTT (20 μL, 5 mg/mL) was added for 4 h, the supernatant was removed, and 100 μL DMSO was added to each well. The optical density (OD) values were obtained at 570 nm.

### 2.7. Cytokine and Enzyme Activity Analyses

The cytokine expression levels and enzyme activities (GSH-Px, SOD) were determined using the respective kits, according to the commercial instructions. The samples were handled according to the introductions for each kit, and the OD values were calculated using a full-wavelength microplate reader.

### 2.8. Western Blot Analysis

Protein lysates were added to tissue homogenates and total protein for each sample was extracted by centrifugation. Total protein concentrations were tested using a Bicinchoninic Acid (BCA) kit, then denatured protein samples were used for subsequent studies. Protein samples were separated on 10% SDS-PAGE, transferred to PVDF membranes (Millipore, Burlington, MA, USA), and blocked with 5% skim milk at room temperature for 2 h. The membranes were then incubated with specific primary antibodies (1:1000 dilution) overnight. Finally, the membranes were incubated with secondary antibodies (1:3000 dilution) and determined using ECL chemiluminescence reagent.

### 2.9. qRT-PCR Assay

Total RNA in mMECs was extracted using Trizol reagent (Invitrogen, Carlsbad, CA, USA) and then converted into cDNA using a reverse transcription kit (Takara, Otsu, Japan). The primers (*Nrf2* and *GAPDH*) were designed using primer 5.0 software (Premier company, Canada) and are shown in Table 1. *GAPDH* was used as an internal standard. Relative fold changes in gene expression levels were calculated using the 2^−ΔΔCt^ comparative method.

### 2.10. Network Pharmacological Analysis

The pharmaceutical property of SG was estimated using network pharmacology technology. The Swisstarget website was used to predict the potential of SG, and metascape software (https://metascape.org/gp/index.html#/main/step1, accessed on 9 September 2022) was used to analyze the target genes via GO and KEGG. Finally, Cytoscape software provided a visual of the SG targeting pathway network.

### 2.11. Immunofluorescence Analysis

Paraffin slices were immersed in xylene for dewaxing and were dehydrated with ethanol at different concentrations along a gradient. The tissue slices were permeated with PBS appending Triton X-100 (Sigma, San Diego, CA, USA) and 10% BSA, then incubated overnight with special primary antibodies and corresponding secondary antibodies. Nuclei were stained with DAPI reagent. Finally, all sections were observed under a fluorescence microscope.

### 2.12. ROS Analysis

The production of ROS in mMECs was determined using an ROS Assay Kit (Beyotime, Hangzhou, China). Cells (1 × 10^5^ cells/mL) were passed into 6-well plates and then incubated with control media or LPS in the presence or absence of SG (5, 25, and 50 µM). The cells were incubated with DCFH-DA for 1 h in the dark, and extracellular DCFH-DA solution was removed. Finally, relative levels of fluorescence were quantified using a fluorescence plate reader MTP902 (Olympus, Tokyo, Japan).

### 2.13. Data Analysis

Statistical analysis was conducted with SPSS software. The results are presented as means ± SDs. All data in the present study were analyzed by one-way ANOVA followed by Dunnett’s test, and significant differences were determined at *p* < 0.05.

## 3. Results

### 3.1. Network Pharmacological Analysis of SG

**Figure 2:**
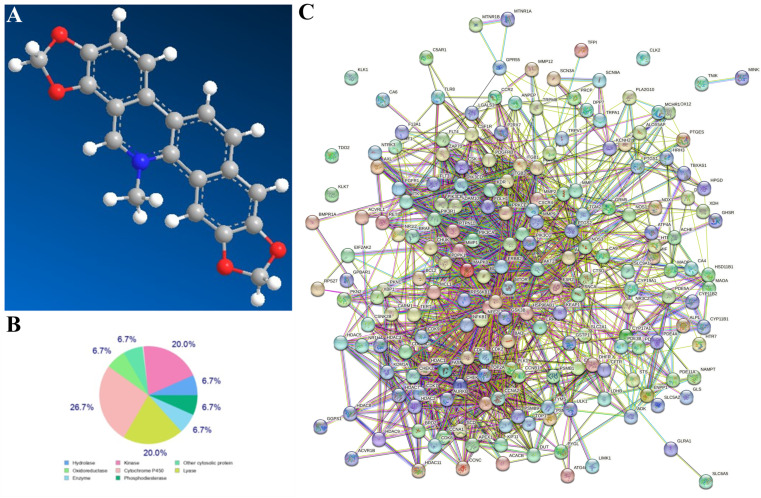

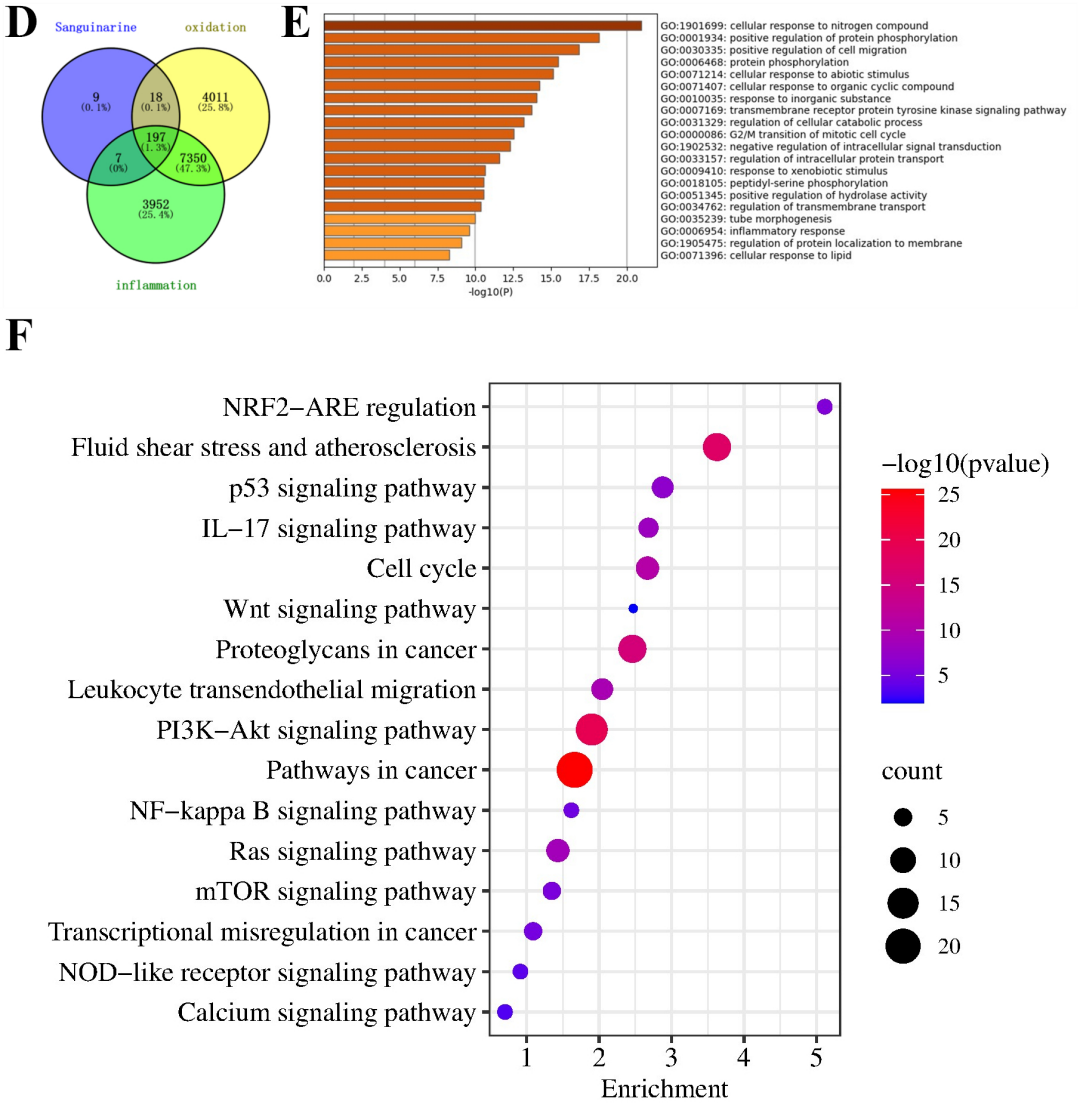
Network pharmacological analysis of SG. (**A**) Three-dimensional structure formula of SG. (**B**) The target classes of SG. (**C**) The potential targets of SG were predicted using the SwissTarget website. (**D**) The common target genes in “SG”, “inflammation”, and “oxidation”. (**E**,**F**) GO annotation and KEGG were used to analyze these target genes.

The development of bioinformatics technology, especially network pharmacological analysis, allowed for more accurate predictions in this experiment. The results showed 197 common genes in “SG”, “inflammation”, and “oxidation”. GO annotation and KEGG analysis showed that these target genes were related to oxidative stress and inflammatory response (Figure 2).

### 3.2. Cell Viability and Biological Assay

**Figure 3:**
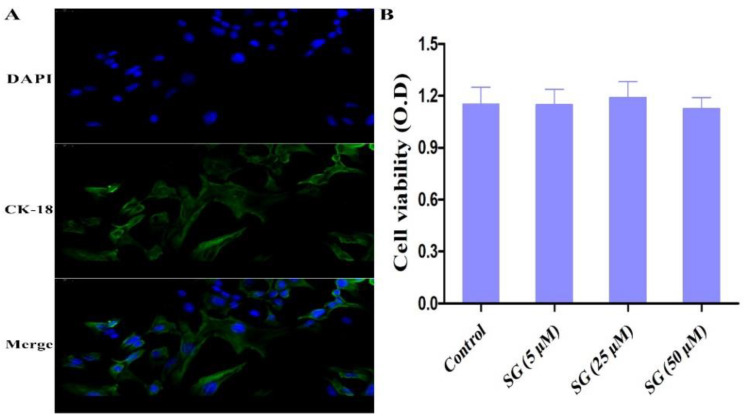
Cell viability and biological detection. (**A**) Cytokeratin-18 was used to identify the integrity of mMECs (scale bar: 20 µm). (**B**) The effect of SG on cell viability was detected by MTT assay. Data are presented as the means ± SEMs of three independent experiments.

Cytokeratin-18 was used to identify the integrity of mMECs (Figure 3A). The cell viability of mMECs was assessed by MTT assay. As shown in Figure 3B, the cell viability of mMECs was not affected by the SG treatment.

### 3.3. Effect of SG on LPS-Induced Oxidative Stress

**Figure 4:**
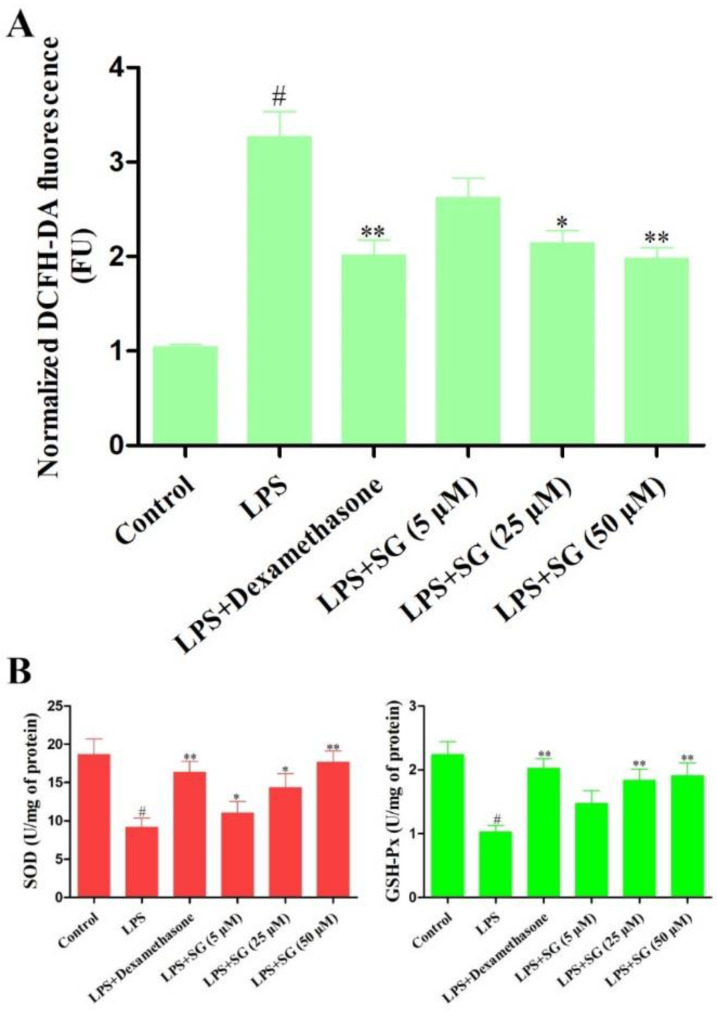
Effect of SG on LPS-stimulated oxidative stress. (**A**) Effect of SG on LPS-triggered ROS production in mMECs. (**B**) The activities of anti-oxidative enzymes were determined using commercial kits. Data are presented as the means ± SEMs of three independent experiments. The symbol # indicates *p* < 0.05 vs. the control group. The symbols * and ** represent significant differences at *p* < 0.05 and *p* < 0.01, respectively.

The increase in ROS yield caused by LPS was significantly alleviated under SG treatment (Figure 4A). In addition, the enzyme activities of superoxide dismutase (SOD) and glutathione peroxidase (GSH-Px) were also determined using commercial kits (Jiancheng Bioengineering institute, Nanjing, China) in LPS-stimulated mMECs. The results showed that the enzyme activities of SOD and GSH-Px in the LPS challenge group were lower than those in the control group, but SG significantly increased the activities of SOD and GSH-Px (Figure 4B).

### 3.4. SG Alleviated LPS-Induced Mammary Gland Injury in Mice

**Figure 5:**
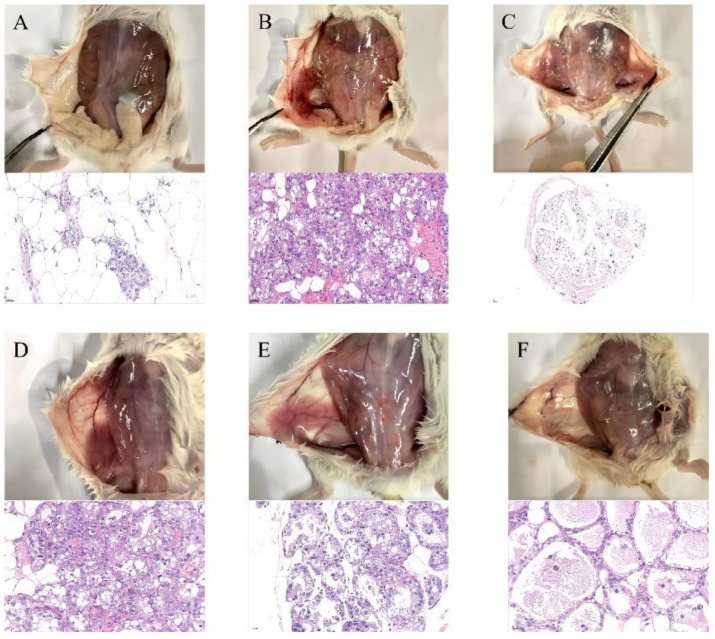
SG alleviated LPS-induced mammary gland injury in mice (HE; scale bar: 50 µm). (**A**) Control group. (**B**) LPS group. (**C**) LPS + dexamethasone group. (**D**–**F**) LPS + SG groups (5, 25, and 50 µM).

Histological changes in mouse mastitis stimulated by LPS were evaluated by H&E staining. Morphological changes in mammary glands were observed after the LPS and SG treatments (Figure 5). The results of the histopathological analysis suggested that, compared with the control group, LPS caused obvious pathological changes, including breast tissue congestion, extensive inflammatory cell infiltration, and destruction of acinar structures (Figure 5A,B). However, severe histopathological changes induced by LPS were greatly attenuated by dexamethasone or SG treatment, especially at high concentrations of SG (Figure 5C–F).

### 3.5. SG Reduced LPS-Induced Inflammatory Response and Improved the Integrity of the Blood–Milk Marrier

**Figure 6:**
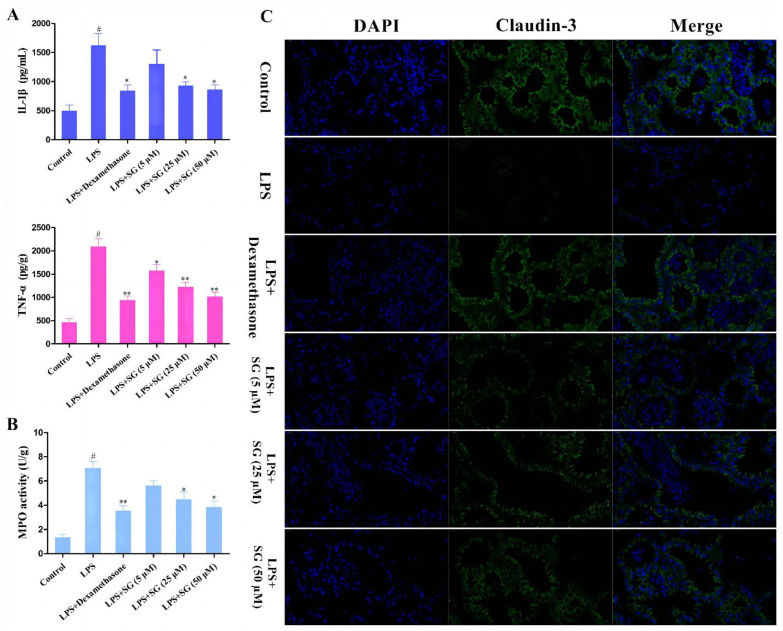
SG reduced LPS-induced inflammatory response and improved the integrity of the blood–milk barrier. (**A**) The expression levels of TNF-α and IL-1β in tissues were detected by ELISA assays. (**B**) MPO activity. (**C**) The tight junction protein Claudin-3 was detected by immunofluorescence assay (Scale bar: 20 µm). Data are presented as the means ± SEMs of three independent experiments. The symbol # indicates *p* < 0.05 vs. the control group. The symbols * and ** represent significant differences at *p* < 0.05 and *p* < 0.01, respectively.

It is well known that TNF-α and IL-1β play vital roles in inflammatory response. In order to analyze the effect of SG on LPS-induced inflammation, the expression levels of TNF-α and IL-1β in tissues were detected by ELISA assays. LPS stimulation could markedly increase the expression of TNF-α and IL-1β. Compared with the LPS group, SG treatment greatly decreased the levels of these pro-inflammatory cytokines (Figure 6A). Moreover, myeloperoxidase (MPO) is a heme protein rich in neutrophils and serves as a marker of neutrophil function and activation [21]. The results showed that SG treatment significantly reduced LPS-induced MPO activity (Figure 6B). The tight junction proteins, such as Claudin-3, play vital roles in the blood–milk barrier [23]. An immunofluorescence technique was used to evaluate the integrity of the blood–milk barrier. The results showed that SG significantly reduced the inhibition by LPS of the expression of the tight junction protein claudin-3 (Figure 6C).

### 3.6. Effects of SG on the Activation of Nrf2 and the Wnt/β-Catenin Pathway

**Figure 7:**
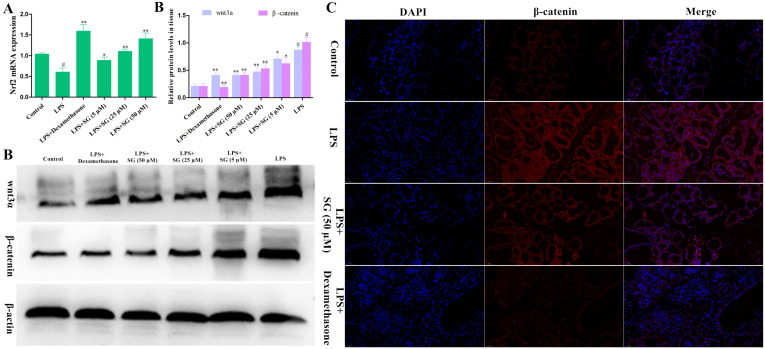
Effects of SG on the activation of Nrf2 and the Wnt/β-catenin pathway. (**A**) The expression of Nrf2 was determined by qRT-PCR assay. (**B**) The levels of proteins in the Wnt/β-catenin pathway were detected by Western blot assay. (**C**) The level of β-catenin protein was determined by an immunofluorescence technique (scale bar: 50 µm). Statistical analysis of the Western blot quantification should be carried out by performing a multiple *t*-test. Data are presented as the means ± SEMs of three independent experiments. The symbol # indicates *p* < 0.05 vs. the control group. The symbols * and ** represent significant differences at *p* < 0.05 and *p* < 0.01, respectively.

It has been found that the activation of Nrf2 is related to oxidative stress and inflammatory reaction [24]. An immunofluorescence technique was used to detect the expression levels of Nrf2 protein in the mammary gland tissues. As shown in Figure 7A, SG treatment could significantly increase the activation of Nrf2, but the activation of Nrf2 was reduced by LPS challenge. Additionally, the Wnt/β-catenin signaling pathway plays a crucial role in LPS-induced inflammation [25]. Thus, we also investigated the effect of SG on the Wnt/β-catenin pathway in LPS-induced mouse mastitis. Compared with the control group, LPS challenge significantly increased the expression of wnt3a and β-catenin proteins. In contrast, SG treatment significantly reduced the expression levels of wnt3a and β-catenin (Figure 7B).

## 4. Discussion

Mastitis is a common clinical disease in dairy cows, which affects the health and welfare of dairy cows and causes huge economic losses to the dairy industry [26,27]. At present, the most commonly used treatment for cow mastitis is antibiotic therapy. However, the nonstandard use of antibiotics leads to drug resistance and drug residues of pathogenic bacteria, which bring greater challenges in the prevention and treatment of mastitis and affect the quality of dairy products [28]. Therefore, it is imperative to reduce the use of antibiotics clinically, and it is urgent to find new drugs to treat mastitis.

SG has been proved to have anti-inflammatory and anti-oxidative-stress effects, with few side effects [29]. We tried to explore the protective role of SG against mastitis in mice and the mechanisms involved. mMECs are the first line of defense for contacting, recognizing, and responding to foreign microorganisms in the mammary glands, and their role is similar to that of sentinel cells [30,31]. The overproduction of ROS will lead to oxidative stress, which damages the immune and anti-inflammatory functions of dairy cows in the transition period [16]. Moreover, the antioxidant enzymes, such as SOD and GSH-Px, play key roles in the antioxidant defense system of dairy cows [32]. In in vitro studies, SG significantly reduced oxidative stress induced by LPS and increased antioxidant enzyme activity.

One of the characteristics of immune response is the release of cytokines, which play an important role in host immune response to infection and disease [33]. It was reported that IL-1β and TNF-α expression levels were critical to the body’s immunity, but excessive secretion caused fatal systemic inflammation and damaged breast tissue and cells [34]. SG treatment could down-regulate the LPS-induced production of IL-1β and TNF-α. MPO is a biomarker of neutrophil infiltration, can produce reactive oxidants and diffuse free radicals, and is involved in the immune regulation of inflammation. In the process of inflammation, the activity of MPO increases, which can lead to acute and chronic vascular tissue damage [35]. The present experiments found that the mice in the LPS-treated group exhibited significantly increased MPO activity, but the MPO activity gradually decreased with the increase in SG concentration. The above results indicated that SG could protect against the LPS-induced inflammatory injury process by reducing oxidative stress and improving antioxidant enzyme activity.

Nrf2 is an important antioxidant transcription factor, which can reduce inflammation by promoting the expression of its downstream anti-inflammatory genes [36]. In the present study, it was found that SG promoted the expression of Nrf2. Studies have shown that the Wnt/β-catenin signaling pathway is associated with a variety of diseases, including inflammation [37]. Wnt proteins are a family of secreted adiponectins that play decisive roles in cell proliferation, migration, and differentiation [38]. The Wnt/β-catenin signaling pathway could also promote the expression of cytokines and thus aggravate inflammatory response [39]. The present study showed that SG inhibited the LPS-induced activation of the Wnt/β-catenin signaling pathway.

## 5. Conclusions

In conclusion, this study clarified the protective effect of SG against mastitis and provided evidence for new potential mechanisms. The dosage of SG used in this experiment was non-toxic to mMECs. SG not only inhibited the increase in ROS induced by LPS, but also enhanced the activities of antioxidant enzymes. Thus, SG exerted its anti-inflammatory and antioxidant functions by activating Nrf2 and inhibiting the Wnt/β-catenin pathway, repairing the blood–milk barrier.

## Figures and Tables

**Table 1:** Primers used for qPCR.

Name	Sequence (5′→3′): Forward and Reverse	GenBank Accession No.	Product Size (bp)
*Nrf2*	GACCTAAAGCACAGCCAACACAT CTTCAATCGGCTTGAATGTTTGTC	NM_010902.5	182
*GAPDH*	CAATGTGTCCGTCGTGGATCT GTCCTCAGTGTAGCCCAAGATG	NM_001289726.1	124

## Data Availability

All data are contained in the manuscript.
